# Secretory Carcinoma of the Philtrum of the Upper Lip: A Case Study

**DOI:** 10.7759/cureus.68823

**Published:** 2024-09-06

**Authors:** Jessica Asuquo, Cyril Blavo, Rabi Bhatta

**Affiliations:** 1 Department of Clinical Education, Nova Southeastern University Dr. Kiran C. Patel College of Osteopathic Medicine, Davie, USA; 2 Department of Preclinical Education, Nova Southeastern University Dr. Kiran C. Patel College of Osteopathic Medicine, Davie, USA; 3 Internal Medicine, Southwest Healthcare Medical Education Consortium, Temecula, USA

**Keywords:** etv6-nrt3, histopathology, oncology, secretory carcinoma, submandibular lymphadenopathy

## Abstract

The parotid, submandibular, and sublingual salivary glands are the major salivary glands in the mouth. Cancers that arise in these glands are relatively uncommon, usually benign, and rarely metastasize. We present a unique case of a 17-year-old male diagnosed with high-grade secretory carcinoma of the salivary gland that was generally asymptomatic except for a persistent rash. The patient reported no significant past medical, family, or social history. A multidisciplinary team efficiently diagnosed and treated the cancer with histopathology, MRI, tumor excision, lymphadenectomy, and adjuvant treatment. Despite the rarity of the cancer, which was found in a high-grade regionally advanced disease, in an uncharacteristically young patient, the patient was effectively treated with adjuvant chemoradiotherapy without treatment-related complications.

## Introduction

Secretory carcinoma of the salivary gland (SCSG) is a rare and usually low-grade malignancy predominantly involving the parotid gland, which typically has a good prognosis [1,2]. It often presents as a low-grade tumor diagnosed through clinical presentation, ultrasonography, CT/MRI, and/or biopsy [2,3]. This tumor is characteristically hypoechoic, ovoid, poorly defined, or lobulated [3,4]. It clinically presents as a slow-growing, painless mass of the parotid gland, upper lip, or buccal mucosa that rarely invades surrounding tissues [5,6]. The parotid gland is the most common primary site of tumor growth and masses can appear cystic, solid, or a combination of both [1,7-11]. Distant metastasis may include cervical lymph nodes, lungs, liver, and the brain [3,9]. High-grade aggressive metastasis is uncommon [8].

The diagnostic cellular marker most associated with SCSG is the erythroblast-specific variant 6-neutrophic receptor tyrosine kinase 3 fusion gene (ETV6-NTRK3) [4,5-11]. NTRK3 is a protein-coding gene that transcribes tyrosine kinases, which regulate cell growth and development of the nervous system via neurotrophins [4]. The promoter of the ETV6 gene is far more active than the NTRK3 promoter, so the ETV6-NKT3 [t (12;15)] translocation produces constitutively active tyrosine kinases [5]. Consequently, the overexpression of tyrosine kinases promotes cellular transformation and proliferation, especially in the salivary glands and bone marrow [5]. While there are other cellular markers associated with SCSG, ETV6-NKT3 is largely suspected to be the cause of the oncogenesis [1].

Many cases of SCSG are initially diagnosed as acinic cell carcinoma (AciCC) or pleomorphic adenomas, particularly because they are both histologically and microscopically analogous [7,9]. Immunohistochemistry and genetic molecular testing are the key diagnostic tests that detect the cell markers that differentiate SCSG from AciCC. These cellular markers are S100, GATA-3, AE1/AE3, SOX10, and vimentin [10,11]. The ETV6-NTRK3 fusion gene is not usually found in AciCC, pleomorphic adenomas, or other salivary gland tumors [7,9].

Clinically, SCSG typically presents in adults at about the age of 45 years, as a slow-growing, painless mass with indolent behavior [9,10]. These tumors are usually seen at a low TNM stage of diagnosis and infrequently transform into high-grade aggressive metastasis [8]. However, they can be associated with local/regional lymph node spread [1,10]. Metastasis to the pleura of the lungs, and a manifestation of the rare tumor cell marker, Ki-67, proliferation, are associated with poor outcomes [3,5,9]. While these tumors predominantly occur in the parotid gland of adult men, a vast range of ages of onset have been recorded [1,2,6].

The treatment approach for SCGC and AciCC is surgical resection, which may be followed by adjuvant radiotherapy or molecular-targeted therapy in the setting of high-risk factors [7]. High-risk factors include advanced clinical stage, positive tumor margins, nerve infiltration, extra-nodal extension, and lymphatic and/or vascular metastasis [7]. The occurrence of disease progression is often associated with cervical lymph node metastasis [3], so the identification of the ETV6-NTK3 gene and complete macroscopic resection with local lymph node excision is often recommended [6]. Complete parotidectomy is recommended if the tumor invades the main parotid duct [1]. If the tumor is appropriately distant from the main parotid duct, then localized parotid gland resection with preservation of a portion of the gland is considerable. Due to its path through the parotid gland, facial nerve injury is a probable surgical complication. Cervical lymph node involvement and extra-parenchymal glandular invasion are associated with a greater risk of metastasis and recurrence [1]. Therefore, lymph node excision is recommended in these cases [1,9-10]. Patients may also benefit from targeted biological treatment utilizing tyrosine kinase inhibitors [6].

This article was previously presented as a poster presentation at the 2023 Florida Osteopathic Medical Association Student, Intern, Resident, and Fellow Annual Research Poster Competition on February 3, 2023.

## Case presentation

This is a case of a 17-year-old male who presented at an otorhinolaryngology clinic with a swollen upper lip and erythematous, draining pustules on his philtrum. Symptoms began one year ago when he noticed a painful vesicle approximately 2-3 mm in size, without evidence of drainage or erythema. There was no history of trauma to his lip, and he was not aware of any arthropod bite to his face or lips. Over the period of one year, the vesicular lesion developed into five pustules at about the same site on his upper lip. He characterized the pustules as constantly painful, itchy, and red, while he denied any episodes of cough, hemoptysis, decreased appetite, weight loss, or hemorrhage from the lesions. However, he did admit to recurrent monthly episodes of moderate fevers, which were relieved with doses of acetaminophen. He had no significant past medical, surgical, or family history relevant to this illness.

Physical examination of his lips revealed a diffusely inflamed and edematous upper lip with five inflammatory nodular-pustular lesions with clear, mucoid drainage at the infra-nasal and philtrum region. His head, neck, and general skin examination were unremarkable.

**Figure 1:**
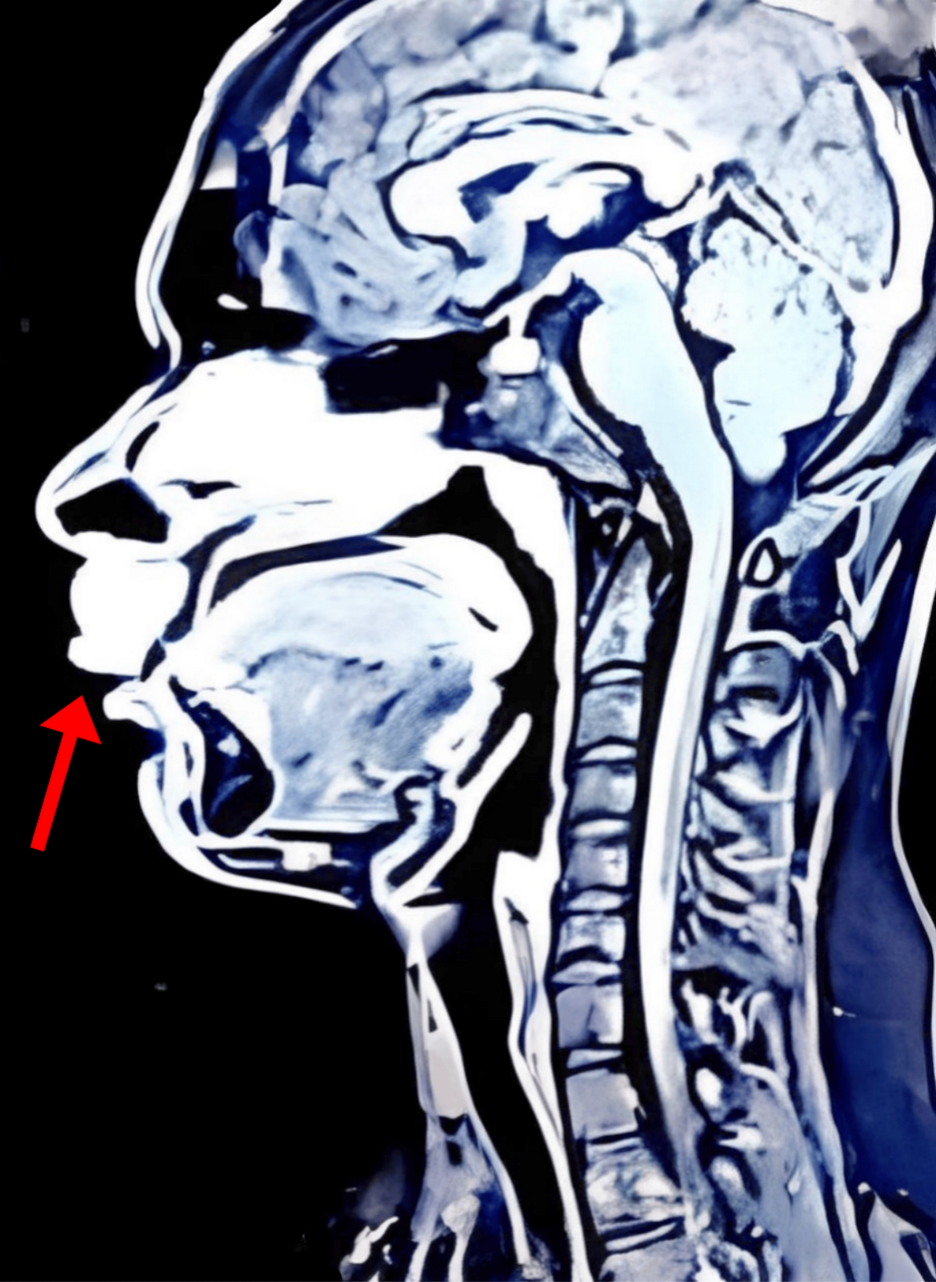
Preoperative sagittal magnetic resonance imaging (MRI)

The patient was prescribed topical mupirocin and oral cephalexin for a week without improvement. Subsequently, a diagnostic workup included ultrasound imaging of his upper lip, which showed hypoechoic lesions with normal vascularity, consistent with benign lesions. Histopathology of the philtrum specimen revealed a high-grade secretory carcinoma with minimal intramural and peritumoral lymph node infiltration. Magnetic resonance imaging (MRI) with contrast showed approximately 25 x 24 x 20 mm irregularly sized, heterogeneously hyperintense lesions suggestive of a malignancy (Figure 1). The lesion invaded the superior orbicularis oris muscle, subcutaneous tissue, and skin of the philtrum with protrusion into the left nostril while bordering the left maxilla. The MRI also indicated bilateral submandibular lymphadenopathy.

**Figure 2:**
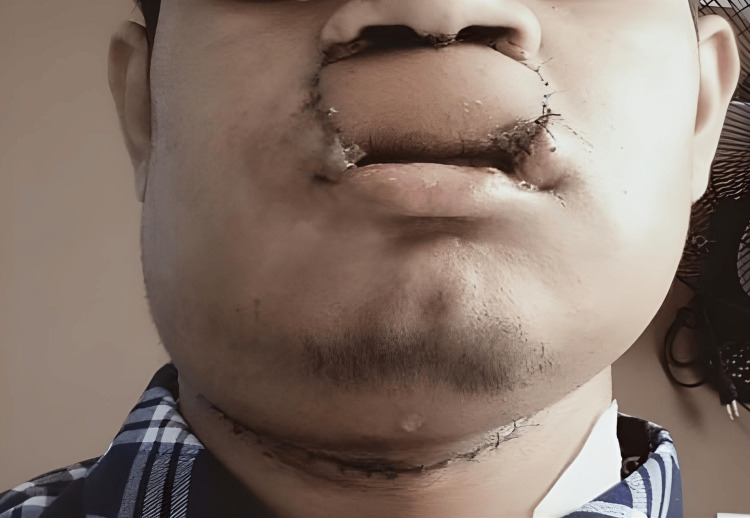
Picture of the patient post-operation

A surgical excision of his upper lip lesion was performed along with a bilateral selective neck dissection (Figure 2). A radial artery free flap was raised for the closure of the defect. Lymph node metastasis was present, but perineural invasion was not identified. The pathological staging of the tumor was pT2N3bM0. In total, 26 lymph nodes were excised, two of which were positive with extra-capsular invasion.

The patient’s post-operative treatment consisted of 30 concurrent sessions of radiotherapy and chemotherapy with cisplatin. Cisplatin was given intravenously at a rate of 30 mg/m^2^ along with concurrent radiotherapy. He tolerated the treatments well except for an occasional epistaxis. Monthly follow-up with CT scans demonstrated no tumor reoccurrence. Notably, at the beginning of radiotherapy treatment, the patient experienced temporary difficulty eating due to tongue paresthesia that resolved after radiation completion. At the six-month follow-up, he demonstrated no new signs of radiotoxicity and was making good progress. He then went to a larger medical facility in India for follow-up care.

## Discussion

Current research suggests that the gold-standard tool to identify and differentiate SCSG from other salivary gland cancers is immunohistochemistry (IHC) paired with genetic sequencing [7]. If these tests identify the presence of the ETV6-NTRK3 mutation, then clinicians have a high probability of accurately identifying SCSG [7,8]. Due to limited hospital resources, genetic sequencing of the punch biopsy was not done. These resources could not only differentiate SCSG from AciCC but can also contribute to more specific drug therapies.

Once a salivary gland tumor is identified as ETV6-NTRK3 positive, the tumor can be classified as SCSG (rather than mucoepidermoid carcinoma, AciCC, or polymorphous adenocarcinoma), and then treatment with TRK inhibitors can be initiated. The literature recommends that larotrectinib and entrectinib, two TRK inhibitors that target the NTRK3 gene, should be the systemic therapy of choice for SCSG [7-9]. However, these newer medications tend to be more costly and therefore less accessible.

In this case, an accurate diagnosis of a rare cancer in a locally advanced form was made by physicians with knowledge and expertise in histopathology. Unfortunately, in areas without advanced pathology labs and diagnostic technology, adequate diagnosis, and treatment of SCSG can be far less likely. Therefore, this case study is a unique example of the identification and treatment of a rare cancer in an uncommon metastatic form.

## Conclusions

This case demonstrates a deliberate and methodical diagnosis and treatment of an uncommon metastatic secretory carcinoma. The pruritic nodules of the patient’s upper lip philtrum were initially suspected as a result of arthropod bites, but during the course of the workup, an oncological etiology was discovered.

MRI and a punch biopsy were used to make the diagnosis of SCSG without genetic molecular testing and/or IHC, thus demonstrating that a diagnosis of SCSG can be made on the basis of histopathology and patient history. Treatment has so far been well-tolerated, and the patient is still being evaluated for long-term treatment effectiveness every six months. Typically, SCSG has a good prognosis and rarely involves cervical lymph node metastasis. Given their rarity, an ongoing surveillance program can be implemented to evaluate and monitor the genetic characteristics of these tumors and indicate when to consider targeted therapy.
